# ActiveDriverDB: Interpreting Genetic Variation in Human and Cancer Genomes Using Post-translational Modification Sites and Signaling Networks (2021 Update)

**DOI:** 10.3389/fcell.2021.626821

**Published:** 2021-03-23

**Authors:** Michal Krassowski, Diogo Pellegrina, Miles W. Mee, Amelie Fradet-Turcotte, Mamatha Bhat, Jüri Reimand

**Affiliations:** ^1^Nuffield Department of Women’s and Reproductive Health, Medical Sciences Division, University of Oxford, Oxford, United Kingdom; ^2^Computational Biology Program, Ontario Institute for Cancer Research, Toronto, ON, Canada; ^3^Department of Molecular Biology, Medical Biochemistry and Pathology, Universite Laval, Quebec, QC, Canada; ^4^Oncology Division, Centre Hospitalier Universitaire (CHU) de Quebec-Universite Laval Research Center, Quebec, QC, Canada; ^5^Multiorgan Transplant Program, University Health Network, Toronto, ON, Canada; ^6^Division of Gastroenterology & Hepatology, Department of Medicine, University of Toronto, Toronto, ON, Canada; ^7^Department of Medical Biophysics, University of Toronto, Toronto, ON, Canada; ^8^Department of Molecular Genetics, University of Toronto, Toronto, ON, Canada

**Keywords:** post-translational modifications (PTM), genome variation, disease genes, cancer drivers, cell signaling, protein interaction networks, databases

## Abstract

Deciphering the functional impact of genetic variation is required to understand phenotypic diversity and the molecular mechanisms of inherited disease and cancer. While millions of genetic variants are now mapped in genome sequencing projects, distinguishing functional variants remains a major challenge. Protein-coding variation can be interpreted using post-translational modification (PTM) sites that are core components of cellular signaling networks controlling molecular processes and pathways. ActiveDriverDB is an interactive proteo-genomics database that uses more than 260,000 experimentally detected PTM sites to predict the functional impact of genetic variation in disease, cancer and the human population. Using machine learning tools, we prioritize proteins and pathways with enriched PTM-specific amino acid substitutions that potentially rewire signaling networks via induced or disrupted short linear motifs of kinase binding. We then map these effects to site-specific protein interaction networks and drug targets. In the 2021 update, we increased the PTM datasets by nearly 50%, included glycosylation, sumoylation and succinylation as new types of PTMs, and updated the workflows to interpret inherited disease mutations. We added a recent phosphoproteomics dataset reflecting the cellular response to SARS-CoV-2 to predict the impact of human genetic variation on COVID-19 infection and disease course. Overall, we estimate that 16-21% of known amino acid substitutions affect PTM sites among pathogenic disease mutations, somatic mutations in cancer genomes and germline variants in the human population. These data underline the potential of interpreting genetic variation through the lens of PTMs and signaling networks. The open-source database is freely available at www.ActiveDriverDB.org.

## Introduction

Genome-wide sequencing and association studies are rapidly increasing the catalog of human genetic variation such as single-nucleotide variants (SNVs) responsible for phenotypic traits and disease risks (Claussnitzer et al., 2020; Karczewski et al., 2020; The 1000 Genomes Project Consortium, 2015). Sequencing of cancer genomes reveals a complex landscape of somatic variation where a minority of driver mutations enable the oncogenic properties of cells by altering the activity of cancer genes and molecular pathways (Bailey et al., 2018; ICGC/TCGA Pan-Cancer Analysis of Whole Genomes Consortium., 2020; Reyna et al., 2020). Extensive somatic variation found in healthy cells in normal tissues (Blokzijl et al., 2016; Martincorena et al., 2015) adds another dimension of genetic complexity and suggests that populations of cells with distinct genetic makeups are present in every individual. Characterizing the implications of genome variation to cellular and physiological function and disease pathogenesis remains a difficult computational and experimental challenge (Gonzalez-Perez et al., 2013; MacArthur et al., 2014).

Post-translational modifications (PTMs) are core components of signaling networks that expand the functional range of proteins by controlling protein activation, degradation, and protein–protein interactions. PTMs are chemical or polypeptide modifications of amino acids that act as molecular switches. Various enzymes add or remove modifications on substrate proteins or read the modified sites to carry out cellular programs (Pawson, 1995). Signaling networks of PTMs are a major focus of therapy development (Gharwan and Groninger, 2016; Hoeller and Dikic, 2009; Jones et al., 2016). Phosphorylation, acetylation, methylation, and ubiquitination are among the most commonly occurring PTMs in human cells whereas hundreds of classes of PTMs are known (Mann and Jensen, 2003; Montecchi-Palazzi et al., 2008). These PTMs are now routinely mapped using high-throughput techniques and consequently, large public datasets for human proteins are available. Major databases such as PhosphoSitePlus (Hornbeck et al., 2015), UniProt (UniProt Consortium, 2019) and others maintain consistently updated collections of PTM sites derived from high-throughput and focused experimental studies.

PTM sites in human proteins are known to be enriched in somatic driver mutations in cancer genomes (Creixell et al., 2015; Radivojac et al., 2008; Reimand and Bader, 2013; Reimand et al., 2013; Wang et al., 2015) and germline variants implicated in the pathogenesis of human diseases and cancer predisposition (Huang et al., 2018; Li et al., 2010; Reimand et al., 2015). In contrast, PTM sites are depleted of genetic variation in the general human population, indicating the functional importance of conserved PTM signaling and the role of evolutionary constraint (Li et al., 2010; Reimand et al., 2015). Thus, integrative analyses of genetic variation using PTMs is likely to contribute to our understanding of molecular and genetic mechanisms. Besides the amino acid substitutions replacing the central modified residue of a PTM site, a larger class of substitutions affects PTMs by altering the short linear motifs recognized by kinases and other enzymes (Creixell et al., 2015; Reimand et al., 2013; Wagih et al., 2015). For example, the sequence motifs targeted by the ubiquitination system and controlling the degradation of cancer driver proteins are commonly affected by somatic mutations (Martínez-Jiménez et al., 2020; Narayan et al., 2016). As a canonical example of PTM-associated cancer driver mutations, substitutions in the N-terminal phosphosites of the oncogene beta-catenin (CTNNB1) stabilize the protein by disrupting phosphorylation-dependent ubiquitylation (Morin et al., 1997), causing downstream activation of the Wnt pathway and resulting in oncogenesis in diverse cancer types. In a recent study, hotspot somatic substitutions in the splicing factor 3B subunit 1 (SF3B1) at the ubiquitinated residue K700 were shown to abolish ubiquitylation, disrupt its mRNA interactions and cause altered splicing of a subset of transcripts (Zhang et al., 2019), consistent with our earlier analysis (Narayan et al., 2016). As proteomic and genetic datasets grow rapidly, systematic analyses and data resources allow researchers to study potential disease mechanisms involving genetic variation in signaling networks.

We developed the ActiveDriverDB database (www.ActiveDriverDB.org) to facilitate integrative analyses of human genetic variation and PTM sites. We present a major update to our original publication (Krassowski et al., 2017) that includes additional genomic and proteomic datasets, new types of PTMs and improved workflows. We included a phosphoproteomics dataset of SARS-CoV-2 response (Bouhaddou et al., 2020) to enhance integrative analyses of human population variation and infection-specific PTMs. This article describes the major workflows of our database and reviews the recent updates.

## Results

### The ActiveDriverDB Server

**FIGURE 1:**
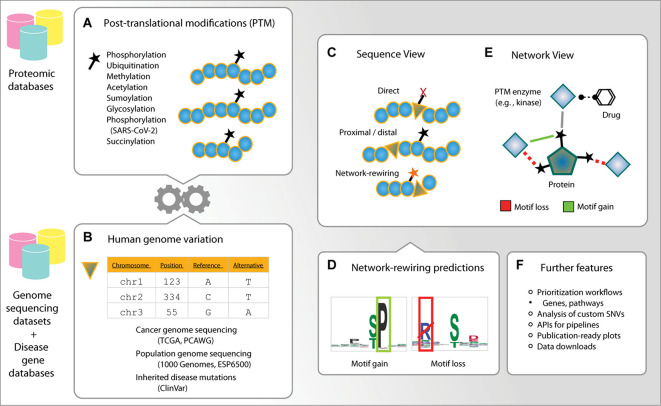
Outline of ActiveDriverDB. ActiveDriverDB is an interactive proteo-genomics database for interpreting human genetic variation using post-translational modification (PTM) sites. **(A,B)** The database integrates PTM sites from experimental studies collected from proteomics databases with amino acid substitutions from genome sequencing projects and curated databeses of disease mutations. **(C)** In the *Sequence View*, substitutions in PTM sites are classified based on their functional impact as direct (at a PTM residue), proximal or distal (within 1–2 or 3–7 positions of a PTM residue), or network-rewiring. **(D)** Network-rewiring substitutions at PTM sites are predicted to disrupt short linear motifs or create new motifs bound by kinases and other enzymes. **(E)** In the *Network View*, proteins and PTM sites are visualized with their interactions with PTM enzymes (e. g., kinases) and the known drugs targeting the enzymes. **(F)** The database also provides prioritized lists of genes and pathways, comprehensive data visualizations and an application user interface (API) for analysing custom variant datasets using computational pipelines.

ActiveDriverDB is a web-based database for interpreting protein-coding variation in human genomes using PTM sites (Figure 1). Our leading hypothesis is that amino acid substitutions caused by SNVs in PTM sites can alter signaling networks by creating, altering, and disrupting the sites. Genetic variation of PTM sites can affect modification and downstream signaling directly by substituting the modified residue or indirectly by modifying the consensus binding sequences (i.e., short linear motifs) located in the flanking sequences of post-translationally modified residues. Thus, an integrated analysis of PTM sites and genetic variation can evaluate the functional impact of variants and lead to mechanistic insights.

**FIGURE 2:**
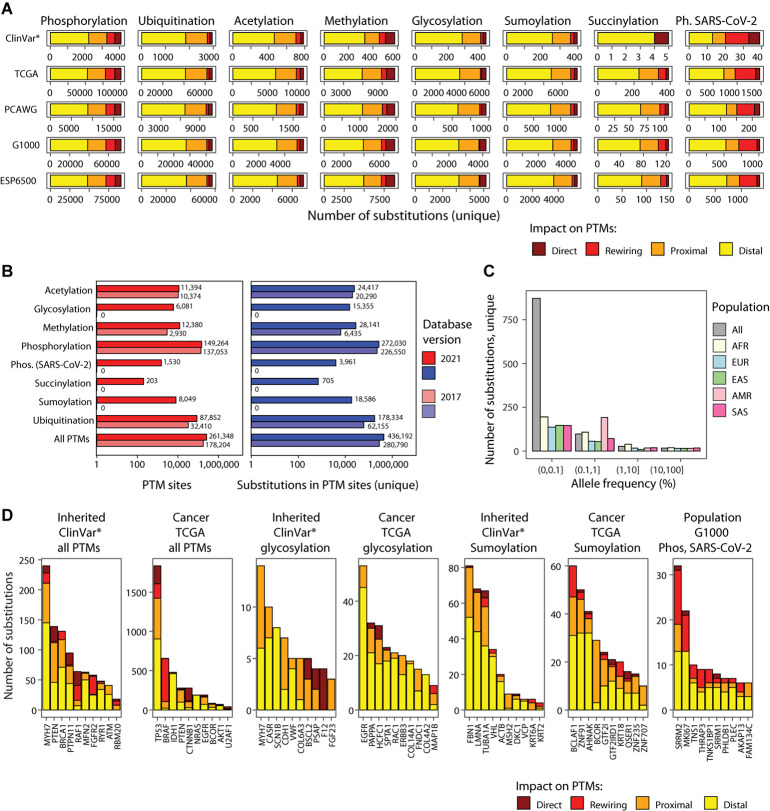
PTM sites and mutations in ActiveDriverDB. **(A)** Summary of genetic variants (i.e., amino acid substitutions) affecting PTM sites in the database. Eight types of PTM sites are shown as horizontal stacked bar plots (left to right) with five genome variation data-stes (top to bottom): interited disease mutations (*ClinVar: only pathogenic and likely pathogenic variants), somatic cancer mutations (TCGA, PCAWG) and human population variation (1000 Genomes, ESP6500). Colors indicate the predicted impact of substitution on PTM sites. Total numbers of unique PTM-associated substitutions in consensus protein isoforms are shown. **(B)** Bar plot shows counts of PTM sites and relatedd substitutions in ActiveDriverDB. The current and previous versions of the database are compared. **(C)** Allele frequency of substitutions in the human population (1000 Genomes) affecting the phosphosites modulated by the SARC-CoV-2 infection in Vero E6 cells. Population cohorts are shown in colors (AFR, African; Admixed American; EAS, East Asian; EUR, European; SAS, South Asian). **(D)** Top genes with PTM-related substitutions in all PTM sites in inheried disease and cancer, genes with glycosylation and sumoylation-associated subtitutions, and top genes in the human population with SARS-CoV-2-specific phosphosites affected by substitutions. Colors indicate the predicted impact of substitutions on PTM sites. Genes were prioritized using ActiveDriver (FDR < 0.05), except for the rightmost group where unique substitution counts were used.

To address this hypothesis, we collected more than quarter of a million unique, experimentally detected PTM sites in the human proteome using the powerful resources available in the public databases PhosphoSitePlus (Hornbeck et al., 2015), UniProt (UniProt Consortium, 2019), Phospho.ELM (Dinkel et al., 2011), and HPRD (Keshava Prasad et al., 2009; Figures 1A, 2A,B). ActiveDriverDB covers seven major types of PTMs with the largest sets of experimental data available for the human proteome. These include 149,299 phosphorylation sites (57%), 87,852 ubiquitination sites (34%), 12,380 methylation sites (4.7%), 11,394 acetylation sites (4.4%), and three types of PTM sites added in the 2021 update of the database: 6,081 glycosylation sites (2.3%), 8,049 sumoylation sites (3.1%), and 203 succinylation sites (0.08%). The 261,348 unique PTM sites occur in proteins encoded by 15,570 genes (i.e., 82% of protein-coding genes). Different types of PTMs are known to act in concert in important cellular processes (Dantuma and van Attikum, 2016). Consistently, a fraction of mutated PTM sites (5.5%) is affected by multiple types of PTMs, suggesting that such complex signaling activities may be altered through amino acid substitutions. In this article, we summarize the counts of PTM sites and substitutions in canonical protein isoforms for individual genes, however, our database includes all high-confidence protein isoforms with 552,068 PTM sites. These data show the extent of PTMs in the human proteome and underline their value in interpreting protein-coding genome variation using our database.

**FIGURE 3:**
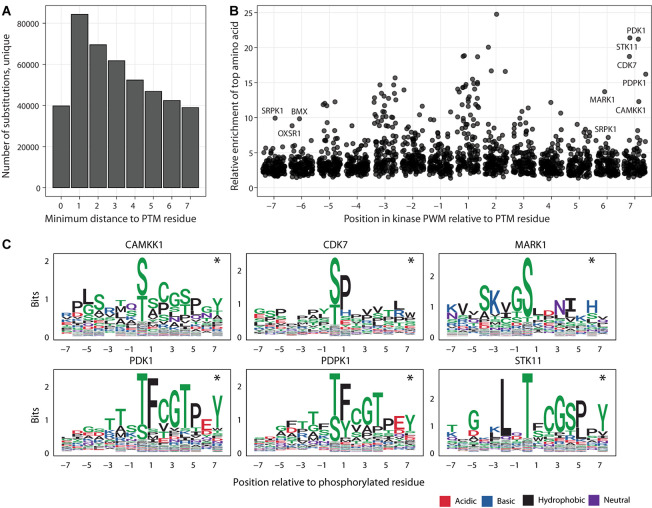
Putative impact of adjacent and distal PTM-flanking residues on kinase binding motifs. **(A)** Histogram of substitutions in PTM sites relative to the distance to the closest modified residues. **(B)** Enrichments of amino acids in the 125 kinase binding site models of position weight matrices (PWMs). Each point represents a position in the consensus binding sequence (short linear motif) of a specific kinase. For each flanking position in the motif (X-axis), the amino acid with the highest enrichment relative to its proteome-wide distribution is shown on the Y-axis, indicating the potential impact of sustitutions at these positions. Kinases with amino acids showing at least eight-fold enrichment at the furthest flanking positions (6th, 7th) are labelled. **(C)** Examples of kinases with enrichments at the 6th and 7th flanking positions of PTM sites. PWM logos show the prevalence of specific amino acids (Y-axis) at the flanking positions (X-axis). Asterisks show the furthest flanking positions from panel A.

We analyzed human genetic variation datasets of three classes using flanking sequences of seven amino acids on both sides of the post-translationally modified residue (Figures 1B, 2A,B). First, we integrated the ClinVar catalog of inherited disease mutations (Landrum et al., 2020) with 237,930 unique amino acid substitutions, of which 65,162 (27%) affected PTM sites. We prioritized 28,976 substitutions classified as *pathogenic* or *likely pathogenic* in ClinVar and found that 6,913 (24%) of these affected PTM sites. When considering the entire ClinVar dataset of disease-associated substitutions, 22% occurred in PTM sites (65,162/237,930). Second, we integrated somatic genome variation of human cancers of nearly 40 types, including the Cancer Genome Atlas (TCGA) PanCanAtlas dataset with ∼10,000 cancer exomes (Ellrott et al., 2018), as well as the ICGC/TCGA Pan-Cancer Analysis of Whole Genomes (PCAWG) dataset with ∼2,500 whole cancer genomes (ICGC/TCGA Pan-Cancer Analysis of Whole Genomes Consortium., 2020) added in the 2021 update of our database. This resulted in a total of 889,792 unique amino acid substitutions, of which 179,470 (20%) affected PTM sites. Third, we integrated two datasets of genome variation in the human population, the 1000 Genomes Project (The 1000 Genomes Project Consortium, 2015) and ESP6500 (Tennessen et al., 2012) with a total of 1,047,196 unique amino acid substitutions, of which 217,932 (21%) affected PTM sites. Together, these genetic maps include 2,049,883 unique amino acid substitutions of which 436,192 (21%) are predicted to affect PTM sites. Our variant impact predictions show the strongest effects on a subset of substitutions in PTM sites: 37,186 (8.5%) substitutions replace the central PTM residue and therefore likely to abolish PTMs, and 35,136 (8.1%) are predicted to create or disrupt kinase-binding motifs by substituting important amino acid residues within seven positions of PTM sites (Wagih et al., 2015). The majority of substitutions are classified as proximal (30%) or distal (53%) and are located at 1–2 or 3–7 positions from the nearest PTM site, respectively (Figure 3A). Most proximal and distal substitutions cannot be interpreted reliably in the context of known kinase-binding motifs; however, these may affect uncharacterized sequence motifs of phosphorylation and other PTM types or cause smaller alterations of sequence motifs (Figures 3B,C). The genomic variation of amino acid substitutions in PTM sites provides a wealth of novel hypotheses for further computational and experimental studies to understand genotype–phenotype associations and PTM function.

#### The Sequence View

The first major workflow of ActiveDriverDB starts with a gene ID of interest provided by the user. The database displays an interactive color-coded overview of the protein sequence where the amino acid substitutions are annotated with respect to their impact on PTM sites and their frequency in the genetic dataset (Figure 1C). The user may choose to focus on cancer genomes, inherited diseases, or genome variation in the human population. The data can be filter based on the disease subtype, type of PTM or the annotations of genetic variants. Four categories are used to classify the PTM-specific impacts of substitutions. *Direct* mutations substitute a central, modified residue of a PTM site with another non-modifiable amino acid residue that will likely disrupt PTMs at the site. *Proximal* and *distal* mutations induce a substitution within 1–2 or 3–7 residues, respectively, from the closest PTM site. For a subset of distal and proximal mutations, we predict that the substitutions have a plausible *network-rewiring* effect since they disrupt an existing short linear motif of a known kinase or other PTM enzyme (i.e., *motif loss*) or create a new sequence motif (i.e., *motif gain*) in the flanking sequence of the PTM site (Figures 1D, 2A). Network-rewiring mutations are predicted using the MIMP method that uses a machine-learning framework of Gaussian mixture models and Bayesian posterior probability estimation to quantify the impact of substitutions on short linear motifs (Wagih et al., 2015). The Sequence View also displays a table of mutations and their impact on PTM sites, information on protein domains (Finn et al., 2017), evolutionary conservation (Pollard et al., 2010) and disorder (Ward et al., 2004), and hyperlinks to external databases. This view allows researchers to construct experimentally testable hypotheses of variant function and associations with phenotypes and disease.

#### The Network View

The second major workflow starts from a gene of interest in a protein–protein interaction network. The network shows the protein as the central node (i.e., the substrate) and all kinases and other PTM enzymes targeting the protein are shown as peripheral nodes. Approved drugs targeting these PTM enzymes, derived from the DrugBank database (Wishart et al., 2018), are displayed via secondary peripheral interactions of the network. The Network View focuses on enzyme–substrate interactions that occur at individual PTM sites and provides predictions of substitutions causing gains and losses of these interactions through altered sequence motifs, derived from the MIMP method (Wagih et al., 2015). Two types of networks are provided. First, the high-confidence *experimental networks* only include experimentally validated enzyme–substrate interactions at specific PTM sites collected from databases and previous studies (Hornbeck et al., 2015; Reimand and Bader, 2013; UniProt Consortium, 2019; Wagih et al., 2015). The lenient *MIMP-predicted networks* include computationally predicted interactions at confirmed PTM sites based on the presence of known kinase binding motifs or *de novo* motifs induced by amino acid substitutions (Wagih et al., 2015). This systems-levels overview of PTM-associated mutations helps predict their impact on downstream signaling networks and discover potential avenues for experimental modulation.

#### Gene and Pathway Prioritization

We statistically analyzed PTM sites and amino acid substitutions to nominate statistically significant cancer driver genes, inherited disease genes, and molecular pathways with enrichments of PTM-associated substitutions (FDR < 0.05), using methods we developed previously (Paczkowska et al., 2020; Reimand and Bader, 2013). The database includes top-ranking genes with frequent PTM-associated mutations in inherited disease and multiple types of cancer (Figure 2D). The genes were prioritized using the ActiveDriver method that uses a Poisson statistical model to identify significant over-representations of substitutions at the PTM sites of individual proteins (Reimand and Bader, 2013). For pathway prioritization, genes with enriched substitutions in PTM sites were collapsed into enriched Gene Ontology terms and Reactome molecular pathways using the ActivePathways data fusion method (Paczkowska et al., 2020). Lists of genes and pathways were derived for the combined set of all PTMs, and also separately for each PTM type. To prioritize genes involved in inherited disease, we focused on the mutations with pathogenic or likely pathogenic effects. Gene and pathway prioritization allows researchers to find individual genes and groups of functionally related genes with PTM-associated disease mutations.

#### Searching, Data Downloads, and Automated Analysis

ActiveDriverDB can be queried interactively and included in automated pipelines. The most common approach is to search the database interactively using a gene symbol or RefSeq ID (e.g., *TP53* or *NM_000345*), or a specific amino acid substitution or a SNV in the GRCh37 version of the human genome (e.g., *IDH1 R132H* or *chr2 209113112 G A*). The database can be queried using names of molecular pathways (e.g., *R-HSA-1640170* or *Cell Cycle*) or diseases (e.g., *Noonan syndrome*) and all genes with such annotations are retrieved. Users can upload a dataset of genetic variants from their experiments to a password-protected area of the database and analyze their data interactively. The upload form supports protein and DNA coordinates of genetic variants. ActiveDriverDB can be used computationally via an Application User Interface (API) of the Representational State Transfer (REST) pattern that provides automated tools to annotate genetic datasets using PTM information. The datasets used in the database are also available for bulk downloads. In this update, we have improved the annotations of PTM sites by adding names of source databases, several classes of protein IDs and flanking sequences of PTM sites. PubMed IDs are available for a subset of sites. The downloadable datasets include PTM sites, PTM-associated substitutions, site-specific enzyme–substrate interaction networks, protein sequences, and disorder predictions. We also provide interactive charts displaying the counts of PTM sites and associated substitutions in the database.

### Genetic Variation in Phosphorylation Sites Induced by SARS-CoV-2 Infection

To enable detailed studies of the cellular changes induced by SARS-CoV-2 infection, we incorporated a recent dataset that quantified the proteome-wide phosphorylation changes in response to SARS-CoV-2 infection in Vero E6 cells of green monkeys (*Chlorocebus sabaeus*) (Bouhaddou et al., 2020). We integrated 1,530 unique SARS-CoV-2 modulated phosphosites in proteins encoded by 949 genes that were detected with significant phosphorylation differences in infected *vs.* control cells at the 24-hour post-infection time point (FDR < 0.05 in infected cells; FDR > 0.05 in controls). The majority of these phosphosites occur on serine residues (88%) followed by threonines (11.3%) and tyrosines (0.7%). We filtered a small subset of phosphosites (1%) that mapped to non-phosphorylatable residues in human proteins (i.e., other than S/T/Y) to avoid inclusion of non-human phosphorylation sites and potential sequence alignment artifacts. This dataset enables integrated analyses of human genome variation, PTM sites and signaling networks underlying the SARS-CoV-2 infection and the coronavirus disease (COVID-19) pandemic.

We evaluated the extent of human genome variation and known disease mutations affecting these phosphosites. ActiveDriverDB includes 3,961 amino acid substitutions affecting SARS-CoV-2-modulated phosphosites. These include 2,007 unique substitutions observed in the two human population cohorts (1000 Genomes; ESP6500) and 1,615 unique substitutions detected in somatic cancer genome sequencing projects (TCGA and PCAWG), and 39 unique substitutions with pathogenic or likely pathogenic effects documented in the ClinVar database (Figure 2A). We evaluated the impact of these PTM-associated substitutions. A relatively large fraction of substitutions (27%) were predicted to create or disrupt kinase binding motifs according to MIMP (Wagih et al., 2015). A minority of substitutions (5.1%) replaced the phospho-residue with another residue, likely causing direct disruptions of signaling. The remaining substitutions were considered as proximal (17%) or distal (51%) relative to the phosphosites. We also studied the allele frequencies of these PTM-specific substitutions in the human population and found that the majority of variants were of low frequency (i.e., less than 1%) in the 1000 Genomes Project dataset (The 1000 Genomes Project Consortium, 2015), however dozens of variants were more prevalent population-wide (Figure 2C). Of the most variable proteins with respect to SARS-CoV-2-specific PTM sites, two are related to alternative splicing (SRRM1, SRRM2) and one to cell cycle regulation (MKI67) (Figure 2D). Interestingly, altered SRRM2 phosphorylation has been also observed in HIV-1 infection (Wojcechowskyj et al., 2013). Collectively, these data suggest that the variable cellular and physiological responses to SARS-CoV-2 infection in humans may have a genetic component that affects the PTM sites and signaling networks that respond to viral infection. Further analysis and experiments may lead to insights to disease mechanisms and therapy options.

### Interpreting Genetic Variation Through Protein Glycosylation

Glycosylation is a type of PTM that involves the conjugation of diverse glycan structures to proteins, in particular extracellular components such as receptors and secreted proteins (reviewed in Moremen et al., 2012; Reily et al., 2019). Glycosylation modifications are conducted by approximately 700 enzymes and multiple subtypes are known, whereas N- and O-linked glycosylation are the most common subtypes. Glycosylation is involved in the folding and quality control of proteins and modulates protein function and protein–protein interactions. Glycosylation of extracellular protein domains in cell–cell signaling contributes to developmental processes and the immune system (Moremen et al., 2012). Aberrant glycosylation patterns, often linked to genetic abnormalities of specific glycosylation enzymes, play important roles in autoimmune diseases such as inflammatory bowel disease, diabetes mellitus, systemic lupus, and congenital disorders of glycosylation (Reily et al., 2019). In cancer, glycosylation is involved in the pathways of metastasis, anti-apoptosis and therapy resistance, and the PTM is also used in diagnostic and prognostic biomarkers (Reily et al., 2019). The increasing availability of comprehensive glycoproteomic datasets generated in human samples (Chen et al., 2009; Liu et al., 2005; Wollscheid et al., 2009) enhances the interpretation of disease genes and mutations using this PTM type.

We collected 7,021 experimentally determined glycosylation sites (including 6,081 unique sites) in proteins encoded by 1,683 genes from proteomics databases (Hornbeck et al., 2015; Keshava Prasad et al., 2009; UniProt Consortium, 2019; Figure 2B). These include the major subtypes of N-glycosylation (2,680 sites) and O-glycosylation (2,856 sites), a few S- and C-linked glycosylation sites, and 1,437 glycosylation sites with no specified subtype. Interestingly, a fraction of proteins (167 or 10%) has glycosylation sites that co-occur with phosphorylation sites, indicating crosstalk of the underlying signaling networks. In total, we found 15,355 unique amino acid substitutions that affect glycosylation sites, including 429 substitutions with pathogenic or likely pathogenic effects in disease genes in the ClinVar dataset and 6,364 somatic substitutions in cancer genomes (Figure 2A). We selected the genes with most significant glycosylation-associated mutations in cancer and inherited disease using ActiveDriver (FDR < 0.05; top 10 genes shown) (Figure 2D). In cancer genomes, frequent substitutions at glycosylation sites are apparent in epidermal growth factor receptors and oncogenes EGFR and ERBB3, as well as PAPPA, a secreted protein involved in the activation of insulin-like growth factor pathways (Lawrence et al., 1999). Germline mutations with pathogenic or likely pathogenic effects at glycosylation sites are associated with cardiomyopathies (MYH7), cancer predisposition (CDH1), epilepsy (SCN1B), and others. These examples showcase an integrative analysis of disease mutations with protein glycosylation sites that may offer insights into disease mechanisms.

### Interpreting Genetic Variation Through Protein Sumoylation

Sumoylation is a PTM that involves the reversible conjugation of SUMO polypeptides (small ubiquitin-related modifiers SUMO1-4) to consensus sequence sites in target proteins (reviewed in Geiss-Friedlander and Melchior, 2007; Flotho and Melchior, 2013; Celen and Sahin, 2020). Sumoylation plays a key role for the cellular response to stress, such as heat shock and DNA damage (Enserink, 2015). In response to DNA damage, sumoylation acts in concert with ubiquitylation events to orchestrate the recruitment of repair proteins to DNA breaks (Dantuma and van Attikum, 2016). A similar interplay of the two modifiers is observed in hypoxic stress response (Cheng et al., 2007). Sumoylation affects lysine residues primarily in nuclear proteins and is thought to regulate protein activation, inactivation and degradation, and protein–protein interactions. Aberrant sumoylation is implicated in malignancies including ovarian, lung, breast, and prostate cancer (Celen and Sahin, 2020; Geiss-Friedlander and Melchior, 2007). Defects in sumoylation are also associated with neurodegenerative pathologies such as Huntington’s, Parkinson’s and Alzheimer’s diseases (reviewed in Yang et al., 2017). Finally, sumoylation is involved in intrinsic and innate immunity and is a target of viral infection (Hu et al., 2016; Liu et al., 2016).

The updated ActiveDriverDB database includes 8,049 experimentally determined sumoylation sites in 2,478 unique genes primarily collected from PhosphoSitePlus (Hornbeck et al., 2015). Interestingly, more than half of sumoylation sites (4,783 or 59%) co-occur with other types of PTMs, in particular ubiquitination sites. We found 19,226 amino acid substitutions at sumoylation sites (16,914 unique), including 8,450 substitutions in the human population genomics datasets, 8,465 somatic substitutions in cancer genomes, and 397 pathogenic or likely pathogenic substitutions of the ClinVar database, suggesting potential disease mechanisms at mutated sumoylation sites. Driver gene analysis of PTM-enriched amino acid substitutions revealed multiple genes with germline and somatic mutations. In the TCGA cancer genomics dataset, the transcription factors (TFs) BCOR (BCL6 corepressor, FDR = 1.2 × 10^–35^) and BCLAF1 (Bcl-2-associated transcription factor 1; ActiveDriver FDR = 9.8 × 10^–4^) were significantly enriched in substitutions in glycosylation sites. Both TFs act as transcriptional repressors of apoptosis and are known as cancer driver genes in the COSMIC Cancer Gene Census database (Futreal et al., 2004). Several other TFs of the less-studied zinc finger family were found in the analysis (Figure 2D). Sumoylation is known as a mechanism of modulating TF activity, thus somatic substitutions in PTM sites may lead to aberrant TF activity in cancer and cause downstream transcriptional deregulation of cancer hallmark pathways. Further study of these substitutions at PTM sites may refine our understanding of known cancer genes and reveal novel candidates.

### Interpreting Genetic Variation Through Protein Succinylation

Succinylation is a PTM that involves the transfer of succinyl groups to lysine residues of substrate proteins via enzyme-dependent and independent means (reviewed in Sreedhar et al., 2020; Trefely et al., 2020). Succinylation has been described only recently (Zhang et al., 2011) and its molecular mechanisms are not fully understood. The highest levels of succinylation are found in mitochondrial proteins, however, high-throughput studies have also detected modifications of cytoplasmic and nuclear proteins. The succinyltransferases CPT1A and KAT2A conduct target-specific modifications while succinyl turnover is controlled by the sirtuin proteins SIRT5 and SIRT7 that regulate bulk succinylation and DNA-damage-dependent succinylation, respectively (Du et al., 2011; Li et al., 2016). The modification is increasingly implicated in transcriptional regulation as histone proteins are often succinylated and site mutations have functional consequences (Smestad et al., 2018; Xie et al., 2012). However, the lysine residues affected by succinylation also undergo other PTMs such as acetylation, methylation and ubiquitylation. Therefore, more research is needed to understand the role of succinylation and its interactions with other PTMs in core cellular processes and human disease (Sreedhar et al., 2020).

Our database includes 203 unique, experimentally determined succinylation sites in proteins encoded by 63 genes, all of which co-occur with other lysine PTMs such as acetylation, methylation, ubiquitylation and sumoylation. Using ActiveDriverDB, we found 772 amino acid substitutions at succinylation sites (705 unique), including 250 substitutions in the human population genomics datasets and 462 somatic substitutions in cancer genomes. In the TCGA cohort of cancer genomes, our analysis highlighted several genes encoding histone proteins (H3J, H2BB, H2BG), reinforcing the role of succinylation in chromatin regulation and suggesting potential PTM-specific driver mutations. In the ClinVar dataset of pathogenic or likely pathogenic mutations, two histone proteins (H3F3A, HIST1H4C) and the copper-zinc superoxide dismutase 1 (SOD1) were highlighted. Mutations in SOD1 are associated with familial amyotrophic lateral sclerosis (Rosen et al., 1993). SOD1 regulates the accumulation of harmful superoxide radicals in cells and coordinated succinylation is required for its function (Lin et al., 2013) whereas mutations impacting its catalytic activity induce the formation of fibrillar aggregates that are toxic for cells (DiDonato et al., 2003). ActiveDriverDB highlights three substitutions flanking the succinylated residue K123 of SOD1 that are annotated as likely pathogenic for amyotrophic lateral sclerosis, suggesting potential hypotheses of these substitutions and altered succinylation in this lethal neurogenerative disease. Further succinylation-associated mutations and putative disease mechanisms are likely to be revealed as larger datasets of these PTM sites are published.

### Improved Annotation of Pathogenic Germline Variants of Human Disease

We updated the collection of inherited disease mutations from the ClinVar database (Landrum et al., 2020) and improved the workflow of interpreting these using PTM sites. The new release of ActiveDriverDB includes 237,930 amino acid substitutions associated with human diseases, a four-fold increase compared to the ClinVar dataset included in the previous version of ActiveDriverDB (56,739). The data have been filtered carefully to only include variants with evidence of involvement in human disease. Genetic variants with germline, parental, maternal, and biparental and *de novo* origin are included in the database while variants of somatic and unknown origin are excluded to improve the analysis of inherited disease variants. Variants can be filtered based on clinical significance (such as *pathogenic*, *benign*, *drug response*, *etc.*) and a star rating reflecting the overall strength of evidence. Hyperlinks to the corresponding records in the databases ClinVar and dbSNP allow researchers to quickly access detailed descriptions of the variants and the original publications reporting the evidence of disease associations and pathogenesis. The updated variant filtering and annotations allow higher-confidence interpretation of disease variants with PTM information.

### Evaluating the Importance of Distal Flanking Residues of PTM Sites Using Sequence Binding Motifs of Kinases

The majority of substitutions in PTM sites in our database are classified as distal and proximal and are located adjacent to modified residues, especially in the three flanking positions (Figure 3A). Only a minority of these substitutions are predicted to have network-rewiring effects since they affect critical sequence residues, however the flanking sequences of PTMs may contain additional functional residues that mediate weaker effects and therefore remain understudied in the database. To quantify the potential effects of proximal and distal substitutions in PTM sites, we systematically analyzed the 130 sequence-binding motifs of kinases used in our database. The motifs are represented as position weight matrices (PWMs) and used for network-rewiring predictions (Wagih et al., 2015). We quantified the PWMs in terms of the strongest amino acid enrichments at each position relative to the proteome-wide distributions of amino acids.

We found that each position of flanking sequence around the PTM sites included at least five-fold enrichment of specific amino acids in several sequence-binding models of kinases (Figure 3B). The strongest enrichments of specific amino acids occurred in the flanking windows of three residues around the modified residue. The three flanking positions are also covered by the most substitutions, indicating widespread genetic effects on PTM signaling. However, further positions upstream and downstream of the modified residue also appeared to encode some information with regards to kinase binding. Even when considering only the furthest positions six and seven of the PTM sites, the motifs of 28 kinases included at least five-fold enrichments of certain amino acids whereas more than ten-fold enrichments were observed for six kinases (CAMKK1, CDK7, MARK1, PDK1, PDPK1, and STK11) (Figure 3C). The effects measured here likely represent an underestimate since the sequence specificities of many PTM enzymes remain unknown. In summary, this analysis suggests that substitutions at both proximal and distal flanking positions around the modified PTM sites may affect signaling networks.

Lastly, we asked whether the inclusion of the furthest flanking positions of six and seven from the PTM sites substantially biased our estimates of PTM-associated substitutions seen in known disease genes, in cancer genomes and the human population. Even when excluding the most distal amino acid substitutions at the flanking positions six and seven, a substantial fraction of all human amino acid substitutions is predicted to affect PTM sites. Using this more conservative estimate, PTM sites are affected by 17% of substitutions overall, including 19% of pathogenic or likely pathogenic substitutions in ClinVar and 22% of all ClinVar substitutions, 16% of somatic substitutions in cancer genomes, and 17% of substitutions in the human population genomics datasets. PTM sites, in particular when including the flanking sequences of seven amino acids, are enriched in disease mutations and negatively selected in the human population (Huang et al., 2018; Li et al., 2010; Reimand and Bader, 2013; Reimand et al., 2013; Reimand et al., 2015). Thus, additional functional substitutions likely exist in the flanking sequences of PTMs that cannot be interpreted yet using current proteomics datasets and computational models.

## Discussion

The increasing availability of genomic and proteomic technologies expedites the development of diverse applications in research, medicine and society. Human cells and tissues can be profiled at an improved resolution and decreased cost and cause an increasing influx of multi-omics datasets in the public domain. The collection of experimentally validated PTM sites in ActiveDriverDB has grown by 47% compared to the first release of the database in 2017 (261,348 vs. 178,204) while the dataset of disease-associated genome variants has quadrupled in size. Thus, we have the opportunity to interpret an ever-larger number of protein-coding variants in the human genome at an enhanced level of detail. In particular, the network-rewiring impact of variants is likely underestimated currently, since high-confidence short linear motifs are known only for a subset of kinases and other enzymes. Careful computational analysis of short linear motifs in conjunction with known PTM sites is required since such low-complexity motifs are statistically expected to occur frequently across the proteome. As we continue to expand the known repertoire of sequence-binding specificities of diverse PTM enzymes, we are increasingly able to predict the precise network-rewiring effects of substitutions in PTM sites observed in disease genes and the human population. Incorporation of protein structural information may further expand the collection of PTM-associated substitutions since linearly distant amino acids may affect PTMs through spatial interactions in the three-dimensional structures (Kamburov et al., 2015; Iqbal et al., 2020; Hu et al., 2021; Porta-Pardo et al., 2015). However, as the community rapidly generates larger and more sophisticated experimental datasets, the databases that use these for downstream analyses should be updated as well, since the analysis of -omics datasets with outdated annotations has detrimental effects on data interpretation (Wadi et al., 2016). In future updates of the database, we aim to specifically expand the genetic variation datasets mapping the human population, cancer genomes and inherited diseases. ActiveDriverDB and similar resources (Hornbeck et al., 2015; Wang et al., 2015; Li et al., 2020; Yang et al., 2019) allow a diverse community of molecular and cell biologists, geneticists and computational researchers to interpret complex genomic variation data using PTM sites and signaling networks and to explore detailed hypotheses of molecular mechanisms. These can contribute to the development of innovative therapies, biomarkers and precision medicine strategies.

## Data Availability Statement

Publicly available datasets were analyzed in this study. Processed data can be found here: https://activedriverdb.org/download/.

## Ethics Statement

Ethical review and approval was not required for the study on human participants in accordance with the local legislation and institutional requirements. Written informed consent for participation was not required for this study in accordance with the national legislation and the institutional requirements.

## Author Contributions

MK developed the software, analyzed the data, and performed the data updates. MK, DP, MM, and JR analyzed the data and prepared the figures. AF-T and JR interpreted the data and reviewed the literature. JR wrote the manuscript with significant input from all co-authors. MB and AF-T contributed to project supervision. JR supervised the project. All authors reviewed and edited the manuscript and approved the final version.

## Conflict of Interest

The authors declare that the research was conducted in the absence of any commercial or financial relationships that could be construed as a potential conflict of interest.
